# Machine Learning-Based Multi-Omics Integration for Identification of Hepatocellular Carcinoma Biomarkers in an Egyptian Cohort

**DOI:** 10.1021/acs.jproteome.5c00741

**Published:** 2025-12-30

**Authors:** Rency S. Varghese, Xinran Zhang, Muhammad S. Sajid, Dina H. Ziada, Habtom W. Ressom

**Affiliations:** Department of Oncology, Lombardi Comprehensive Cancer Center, Georgetown University Medical Center, Washington, District of Columbia 20057, United States; Department of Oncology, Lombardi Comprehensive Cancer Center, Georgetown University Medical Center, Washington, District of Columbia 20057, United States; Department of Oncology, Lombardi Comprehensive Cancer Center, Georgetown University Medical Center, Washington, District of Columbia 20057, United States; Department of Tropical Medicine and Infectious Diseases, Tanta Faculty of Medicine, Tanta University, Tanta 31527, Egypt; Department of Oncology, Lombardi Comprehensive Cancer Center, Georgetown University Medical Center, Washington, District of Columbia 20057, United States

**Keywords:** multiomics approaches, liver cancer, mass spectrometry, machine learning, feature selection

## Abstract

Hepatocellular carcinoma (HCC) ranks among the most common causes of cancer-related deaths globally. The high incidence of HCC is largely linked to chronic hepatitis virus infections, liver cirrhosis, and exposure to carcinogenic substances. Egypt has one of the world’s highest burdens of HCC, with liver cirrhosis from chronic hepatitis C virus (HCV) infection as the primary risk factor. Malignant conversion of cirrhosis to HCC is often fatal in part because adequate biomarkers are not available for diagnosis of HCC in the early stage. Therefore, there is a critical need for more effective biomarkers to detect HCC at an early stage, when therapeutic intervention is more likely to be successful. Multiomics integration has emerged as a powerful strategy to uncover biomarkers and better understand the molecular underpinnings of complex diseases such as HCC. This study summarizes findings from multiple untargeted and targeted mass spectrometry-based analyses of proteins, N-linked glycans, and metabolites performed on blood samples from HCC cases and cirrhotic cohorts recruited in Egypt. Integrative analysis using machine learning methods is performed to identify a panel of multiomics features that differentiates HCC cases from the high-risk population of cirrhotic patients with liver cirrhosis.

## INTRODUCTION

Hepatocellular carcinoma (HCC) ranks among the most common cancers globally and is one of the leading causes of cancer-related deaths. It poses a significant public health challenge, particularly in African and Asian regions. Egypt has one of the world’s highest burdens of HCC, primarily driven by chronic hepatitis C virus (HCV) infection. In Egypt, HCC accounts for nearly 70% of all liver cancers, and its incidence has roughly doubled over the past decade, making it the most common cancer in men and the second most prevalent in women.^1^ In 2018 alone, HCC represented nearly one-fifth of all cancer cases, with liver cancer deaths comprising over 32% of cancer mortality.^2^ Chronic liver disease underlies more than 90% of HCC cases in Egypt. Evidently, liver cirrhosis from HCV infection is the dominant risk factor for HCC in Egypt. Elgharably et al. highlights how mass schistosomiasis campaigns between the 1950s and 1980s propagated HCV transmission via unsafe injection practices, creating a large pool of chronically infected individuals who are now at high risk for HCC.^3^ While the rollout of direct-acting antivirals and aggressive national screening and treatment campaigns have dramatically reduced HCV prevalence in recent years, Egypt continues to face a mounting HCC burden due to its aging, previously infected population and the long lag time between viral cure and cancer development. Therefore, there is a critical need to detect HCC at an early stage, when therapeutic intervention is more likely to be successful.

Multiomics integration has emerged as a powerful strategy to uncover biomarkers for complex diseases such as cancer, heart disease, and diabetes. Unlike traditional single-omics approaches that are limited to one molecular layer, multiomics integration combines data from multiple omics layers. Thus, it provides a more holistic understanding of molecular systems, enabling researchers to identify novel biomarkers, uncover previously hidden biological pathways, and cross-validate findings across different data types.

Multiomics integration strategies typically fall into two categories: knowledge-driven and data-driven methods. Knowledge-driven approaches leverage existing biological databases and prior knowledge to map relationships among molecular features.^4^ In contrast, data-driven approaches identify correlations and shared patterns among multiomics data sets or seemingly uncorrelated multiomics features for accurate disease classification. Recently, deep learning models have demonstrated promise in integrative analysis of multiomics data. For example, MoGCN and MOGONET leverage graph convolutional networks for multiomics integration.^5,6^ Also, DeepLIFT, employs meta-learning for interpretable multiomics analysis and pathway enrichment.^7^ Other deep learning models such as DeePathNet, Pathformer, and MoGCN have demonstrated promise in disease classification and pathway-level interpretation.^6,8,9^ These methods collectively enhance the ability to uncover complex biological insights by integrating diverse omics layers across molecular, cellular, and pathway levels. As multiomics data continue to grow, such integrative and interpretable models will be essential for advancing precision medicine and biomarker discovery.

In this study, we investigate machine learning approaches for integration of multiomics data we acquired by analysis of blood samples from HCC cases and patients with liver cirrhosis recruited in Egypt. The goal is to identify a panel of multiomics features that accurately differentiates HCC cases from high-risk population of patients with liver cirrhosis in Egypt.

## MATERIALS AND METHODS

### Study Cohort

Blood samples from 89 subjects (40 HCC cases and 49 patients with liver cirrhosis) recruited from the outpatient clinics and inpatient wards of Tanta University Hospital (Tanta, Egypt) were analyzed using untargeted and targeted proteomics, glycomics, and metabolomics.^10^ The study protocol was approved by the Tanta University ethics committee.^10–12^ Patient characteristics are summarized in Table 1. Blood was collected by peripheral venipuncture into 10 mL BD Vacutainer sterile vacuum tubes and immediately centrifuged at 1000×*g* for 10 min at room temperature. The supernatant was then transferred and centrifuged at 2500×*g* for 10 min at room temperature. Following aliquoting, serum and plasma samples were stored at −80 °C until analysis. Primary tubes and serum/plasma aliquots were labeled with anonymous code numbers without personal identifiers, and these codes were linked to clinical data in a password-protected database.

### Untargeted Multi-Omics Studies

#### LC-MS-Based Metabolomics.

Serum samples from the 89 subjects were prepared by protein precipitation using acetonitrile with internal standards, followed by centrifugation, drying, and reconstitution. Frozen human serum was thawed at room temperature, and 25 *μ*L was mixed with 1.5 mL of 66% acetonitrile containing two internal standards (debrisquinone, 1 *μ*g/mL, for positive mode; nitrobenzoic acid, 10 *μ*g/mL, for negative mode). The mixture was vortexed, incubated on ice for 10 min, then centrifuged at 10,000*g* for 10 min at 4 °C. The supernatant was collected, dried by speed vacuum at room temperature, and reconstituted in 50 *μ*L of mobile phase (2% acetonitrile with 0.1% formic acid). A 5-*μ*L aliquot was injected onto a 50 × 2.1 mm ACQUITY 1.7-*μ*m C18 reverse-phase column on an ACQUITY UPLC system, using a gradient between solvent A (2% acetonitrile in water with 0.1% formic acid) and solvent B (2% water in acetonitrile with 0.1% formic acid). Chromatographic separation was achieved over 10 min at a flow rate of 0.5 mL/min. The samples were then analyzed by UPLC-QTOF-MS (Waters) in both positive and negative ionization modes using a reverse-phase C18 column and a gradient elution.

Raw LC-MS data were first converted to Network Common Data Form (NetCDF) files using MassLynx (Waters). Peak detection was then performed with the XCMS package (Scripps Center for Metabolomics, La Jolla, CA). Following peak detection in each individual sample, peaks were aligned across samples to calculate retention time (RT) deviations and to compare relative ion intensities. This alignment uses a grouping algorithm based on kernel density estimation to cluster peaks in the *m*/*z* domain. The resulting groups are subsequently used to identify and correct run-to-run RT drift. The obtained data matrix was then used to identify peaks whose ion intensities differed significantly between HCC cases and cirrhotic samples.

In total, 274 unique monoisotopic ion masses showed statistically significant differences, with 158 assigned putative metabolite identities. Putative identifications of the monoisotopic masses were found by searching against four databases (HMDB, METLIN, MMCD, and LIPID MAPS). The identities of several putative compounds were confirmed by comparing their MS/MS fragmentation patterns and retention times with those of authentic standards.^12^

#### GC-MS-Based Metabolomics.

Plasma samples from the same subjects were also analyzed using two gas chromatography–mass spectrometry (GC-MS) platforms: GC-qMS (quadrupole MS) and GC-TOFMS (time-of-flight MS), each with distinct temperature programs and column setups, enabling comprehensive untargeted metabolite detection. Plasma metabolites were extracted from 30 *μ*L of plasma with 1 mL of acetonitrile/isopropanol/water (3:3:2) containing isotope-labeled internal standards (1.25 *μ*g/mL each), vortexed, and centrifuged (14,500*g*, 15 min, RT). The supernatant was split into two 460-*μ*L portions (one per GC-MS platform), dried in a SpeedVac, and stored at −20 °C. For derivatization, dried extracts were oximated with 20 *μ*L of 20 mg/mL methoxyamine hydrochloride in pyridine (80 °C, 20 min), cooled, then treated with 91 *μ*L MSTFA + RI standards (80 °C, 20 min), centrifuged (14,500 rpm, 15 min), and 60 *μ*L of supernatant was transferred to autosampler vials. GC-TOFMS data were processed with LECO ChromaTOF; GC-qMS data with AMDIS,^13^ followed by Mass Profiler Professional for alignment and statistics. Both data sets were additionally analyzed in MetaboliteDetector using calculated RI values for alignment.^14^

Putative metabolite identifications were assigned by spectral matching against the Fiehn and NIST libraries. Statistical analysis identified 27 significantly altered metabolites with a false discovery rate (FDR) < 10%. These included known and novel candidates, such as amino acids, organic acids, and sugars. Notably, pathways related to branched-chain amino acid (BCAA) metabolism, TCA cycle, and energy metabolism were implicated.^11^

#### Proteomics.

Serum samples were depleted using the Agilent Plasma 7 Multiple Affinity Removal Spin Cartridge (Agilent Technologies, Santa Clara, CA, USA). Before trypsin digestion, protein concentration in the depleted serum was measured using the micro BCA protein assay (Thermo Scientific/Pierce, Rockford, IL, USA). Thermal denaturation was carried out at 65 °C for 10 min. Samples were reduced by adding 1.25 *μ*L of 200 mM DTT and incubating at 60 °C for 45 min. The reduced proteins were then alkylated by adding 5 *μ*L of 200 mM IAA and incubating at 37.5 °C for 45 min. A second 1.25 *μ*L aliquot of 200 mM DTT was added and the mixture was incubated at 37.5 °C for 30 min to quench excess IAA. Next, 0.8 *μ*g of trypsin was added (enzyme-to-substrate ratio 1:25, w/w), and the sample was incubated at 37.5 °C overnight, followed by microwave-assisted digestion at 45 °C for 30 min at 50 W. Enzymatic digestion was quenched by adding 0.5 *μ*L neat FA to each sample. The samples were then dried in a speed vacuum and reconstituted in 0.1% FA.

Serum samples were analyzed using a Dionex Ultimate 3000 nano-LC system (Dionex, Sunnyvale, CA, USA) coupled to an LTQ Orbitrap Velos mass spectrometer (Thermo Scientific) equipped with a nano-ESI source. LC-MS/MS was performed on tryptic digests corresponding to 1 *μ*g of protein, derived from 0.2 *μ*L of original serum after depletion and digestion. The LTQ Orbitrap Velos was operated with two scan events. The first was a full FTMS scan from 380 to 2000 *m*/*z* at a resolution of 15,000 (at 400 *m*/*z*). The second was a CID MS/MS scan of precursor ions selected from the first scan, using an isolation width of 3.0 *m*/*z*. The normalized collision energy was set to 35%, with an activation Q of 0.250 and an activation time of 10 ms. CID MS/MS was performed on the five most intense ions from each full MS scan.

Protein identification and quantification were done using MaxQuant (based on ion intensity) and Scaffold (based on spectral count). MaxQuant identified 269 proteins, Scaffold identified 231 proteins. Adjusted p-values (FDR < 0.05) were used to identify 38 statistically significant proteins via MaxQuant and 42 via Scaffold.^15^ These proteins were considered as candidates for targeted quantitation and for pathway analysis signifying involvement in coagulation cascades and immune modulation, both relevant to HCC pathogenesis.

#### Glycomics.

N-glycans were enzymatically released from serum proteins, purified, and labeled to enable sensitive detection. Serum (10 *μ*L) was mixed 1:1 with digestion buffer (20 mM ammonium bicarbonate), denatured at 80 °C for 1 h, then treated with 1.2 *μ*L of 10×-diluted PNGase F to release N-glycans at 37 °C for 18 h. Released glycans were purified by 18 h drop dialysis (500/1000 Da MWCO), reduced with 10 *μ*L borane–ammonium complex (10 *μ*g/*μ*L, 60 °C, 1 h), dried with methanol, and permethylated on NaOH-bead spin columns using DMSO/water and two sequential additions of iodomethane. Permethylated glycans were eluted with 50 *μ*L acetonitrile, dried, and separated on an Ultimate 3000 nano-LC with an Acclaim PepMap C18 column (75 *μ*m × 15 cm, 2 *μ*m, 100 Å, 55 °C) prior to analysis on an LTQ Orbitrap Velos mass spectrometer.

Following data preprocessing, 82 distinct N-glycans detected based on their monosaccharide composition were subjected to both univariate statistical testing and machine learning–based feature selection. Using support vector machine–recursive feature elimination (SVM-RFE), 29 glycans were identified as the optimal subset for distinguishing HCC from cirrhosis. This panel achieved a classification accuracy of 77% and an area under the curve (AUC) of 0.87, with several of the selected glycans also showing significance in univariate tests. The findings reveal disease-associated alterations in glycosylation patterns, aligning with known changes in liver pathology. Although glycomics alone provided moderate classification power, its utility was enhanced when integrated with proteomics and metabolomics data in multiomics analyses.^10^

The results from the above untargeted multiomics studies conducted by analysis of blood samples from the 89 subjects laid the groundwork for subsequent targeted multiomics studies. Specifically, we selected 60 metabolites, 100 proteins, and 82 N-glycans for targeted quantitation based on their significance from our untargeted studies and the literature. These targets are listed in Supplementary Table S1. Results from univariate and multivariate statistical analyses using these three data sets have been previously reported.^10,11,15–17^

### Targeted Multi-Omics Studies

#### GC-MS-Based Metabolomics.

Fifty metabolites from a prior untargeted study were quantified in 89 plasma samples by GC-qMS in SIM mode, using the same sample prep, GC conditions, and RI calibration as the untargeted analysis. Targets were metabolites with significant difference between cases and controls in our untargeted analysis and other related studies. For each analyte, four specific ions were monitored (one quantifier, three qualifiers) with ≥10 ms dwell per ion.

Retention times for high-quality targets were obtained with MetaboliteDetector and compared with the Fiehn library, then refined using an in-house EIC-extraction tool that searched peaks around the expected RT, smoothed traces, corrected baselines, and calculated peak width and AUC. Identification was verified with a stringent spectral-match score combining a weighted dot product and average fragment-ratio metric, plus visual inspection.^18^

MetaboliteDetector provided RTs for 37 of 71 metabolites; library RTs were used initially for the remaining 34. After iterative RT adjustment, 67 of 71 analytes were reliably detected (similarity score >0.7) with <1% missing data. Sixty of these with unique putative IDs are further investigated by integrative analysis.

#### Proteomics.

Targeted quantitative analysis of the selected 100 proteins in 89 serum samples was performed by multiple reaction monitoring (MRM) using a Dionex 3000 Ultimate nano-LC system (Dionex Sunnyvale, CA) interfaced to TSQ Vantage mass spectrometer (Thermo Scientific, San Jose CA). Candidate protein biomarkers identified in untargeted LC–MS/MS (MaxQuant and Scaffold) were carried forward for targeted LC–MRM–MS quantitation. For method development, 1 *μ*L of each sample was pooled and a 3 *μ*L aliquot was analyzed to refine transitions and retention times in Pinpoint by matching to the untargeted data. For each peptide, a 12 min RT window centered on the expected RT was used, and the three most intense transitions were retained; peptides or transitions not observed in the pooled run were removed. The final scheduled MRM method quantified 100 proteins (187 peptides, 561 transitions) with a minimum dwell time of 30 ms per transition.

Predefined precursor and transition ions were monitored to selectively detect the targeted peptides corresponding to each candidate protein, using a chromatogram filter peak width of 10.0 s. MRM experiments were carried out with a cycle time of 5.0 s and a Q1 peak width (full width at half-maximum) of 0.70 Da.

The LC-MRM-MS data were analyzed using Skyline (version 2.5.0.6079).^19^ Andromeda search results were used to match LC-MRM-MS transitions. In Skyline, peptide retention times and integration boundaries were optimized per run and refined across runs to remove interfering regions; when multiple peaks occurred, the peak nearest the scheduled RT center was integrated. Transition intensity was calculated as peak area minus background, and protein abundance was obtained by summing intensities of its quantified transitions.^20^

#### Glycomics.

We used an identical sample preparation workflow for both untargeted profiling and targeted quantitation of serum N-glycans, including their release, purification, reduction, and permethylation. Targeted quantitation of 117 N-glycans, including isomeric forms, was performed by MRM on a TSQ Vantage mass spectrometer (Thermo Scientific, Santa Clara, CA). These targets comprised: (i) N-glycans previously detected in our untargeted glycomics studies, (ii) N-glycans reported as potential HCC biomarkers in earlier studies, and (iii) N-glycans associated with Golgi apparatus function retrieved from the KEGG GLYCAN database. The 117 N-glycans were monitored using 213 transitions (three per glycan), reflecting different adduct forms and charge states. Chromatographic conditions matched those of the untargeted profiling, employing an Ultimate 3000 nano-LC system with the same gradient program. The average cycle time for the 213 transitions was 2.7 s. Of the 117 N-glycans, 82 were selected for further investigation in the integrative analysis.

#### Multi-Omics Feature Selection.

**Figure 1.**
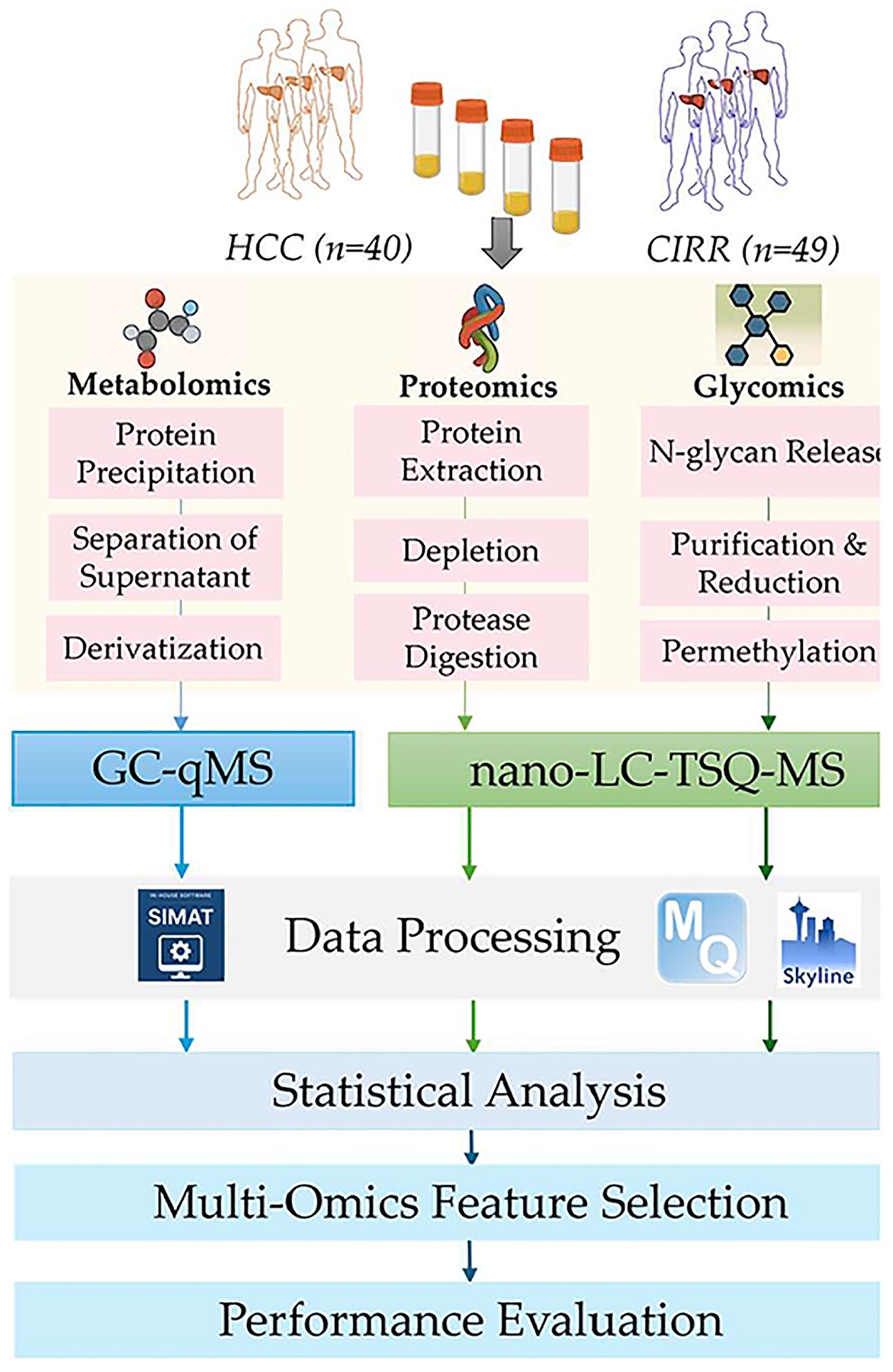
Workflow for integrative analysis of data from targeted multiomics studies.

We investigated integrative analysis of metabolites, proteins, and N-glycans to assess the ability of multiomics features in a panel in distinguishing HCC cases from cirrhotic controls. Figure 1 outlines the workflow of the integrative analysis we performed. As illustrated in the figure, following significant analysis of each omics data set separately using Student’s *t* test, SelectKBest, Elastic Net, SVM-RFE, and Transformer-RFE were applied to rank multiomics features from the combined multiomics data. Additionally, random forest (RF), MOINER, and MOGONET were used first for disease classification by using the combined multiomics data. Then, the multiomics features are ranked based on their contributions for disease classification by using either variable importance in projection (VIP) or SHapley Additive exPlanations (SHAP) values. Finally, the performance of the selected features in disease classification is evaluated.

SelectKBest is a filter-based feature selector in scikit-learn that ranks features with a univariate F-test and returns the K features with the highest scores as the selected subset.^21^

Elastic Net is a supervised feature selection method that combines the penalties of Lasso (L1) and Ridge (L2) regression to perform both coefficient shrinkage and variable selection. During model training, it optimizes a linear regression objective penalized by a weighted sum of the L1 and L2 norms of the coefficients. Features with larger absolute coefficient values are considered more important, while those with coefficients shrunk to zero are excluded from the model. This allows Elastic Net to rank features by the magnitude of their coefficients and retain those most predictive of the output variable.^22^

Support vector machine–recursive feature elimination (SVM-RFE) is a widely used supervised feature selection method that iteratively discards features with the smallest contribution to the SVM classifier, retaining the most informative variables. In SVM-RFE, the estimator (SVM) is first trained on the entire feature set.^23^ The magnitudes of the weight vector serve as feature-importance scores, and the least important features are systematically removed. This process is repeated recursively on the remaining set until a prespecified number of features is selected.

Transformer-recursive feature elimination (Transformer-RFE) is a new feature selection method we developed inspired by SVM-RFE. In this approach, the SVM is replaced with a lightweight cross-attention transformer. The transformer is first trained on the full multiomics data set, and SHAP values computed for the input features are used to derive feature importance scores. After each training run, the feature with the lowest importance score is removed, and the model is retrained on the reduced feature set. This recursive procedure is repeated until a prespecified number of features remains.

Random forest (RF) is an ensemble supervised learning method that builds a collection of decision trees and aggregates their predictions to improve classification or regression performance. Each tree is trained on a bootstrapped subset of the data, and at each split, a random subset of features is considered, which promotes diversity among trees.^24^ This injected randomness helps mitigate overfitting relative to a single decision tree and improves generalization.

MOINER uses a self-attention mechanism to capture correlations among omics features and exploits these relationships for disease classification. Information is enhanced through neighborhood aggregation and message passing over a sample similarity network (SSN), thereby enriching the data representation. A vision transformer (ViT) is then applied for classification. In this way, the method embeds multiomics profiles as images and leverages deep attention architectures to integrate heterogeneous data.

MOGONET first constructs an SSN for each omics modality based on the cosine similarity of feature profiles, and then trains parallel graph convolutional networks (GCNs) to learn view-specific embeddings. These embeddings are fused using the View Correlation Discovery Network (VCDN). By combining omics-specific GCNs with VCDN, the model captures cross-omics correlations in the label space, and final classification is performed via VCDN.^5^

#### Performance Evaluation.

The discriminative performance of the top five features selected by each method was assessed by using them as a panel in a logistic regression model to classify HCC versus cirrhosis. 5-fold cross-validation was applied to estimate the average classification accuracy and the area under the receiver operating characteristic curve (AUC).

## RESULTS & DISCUSSION

**Figure 2.**
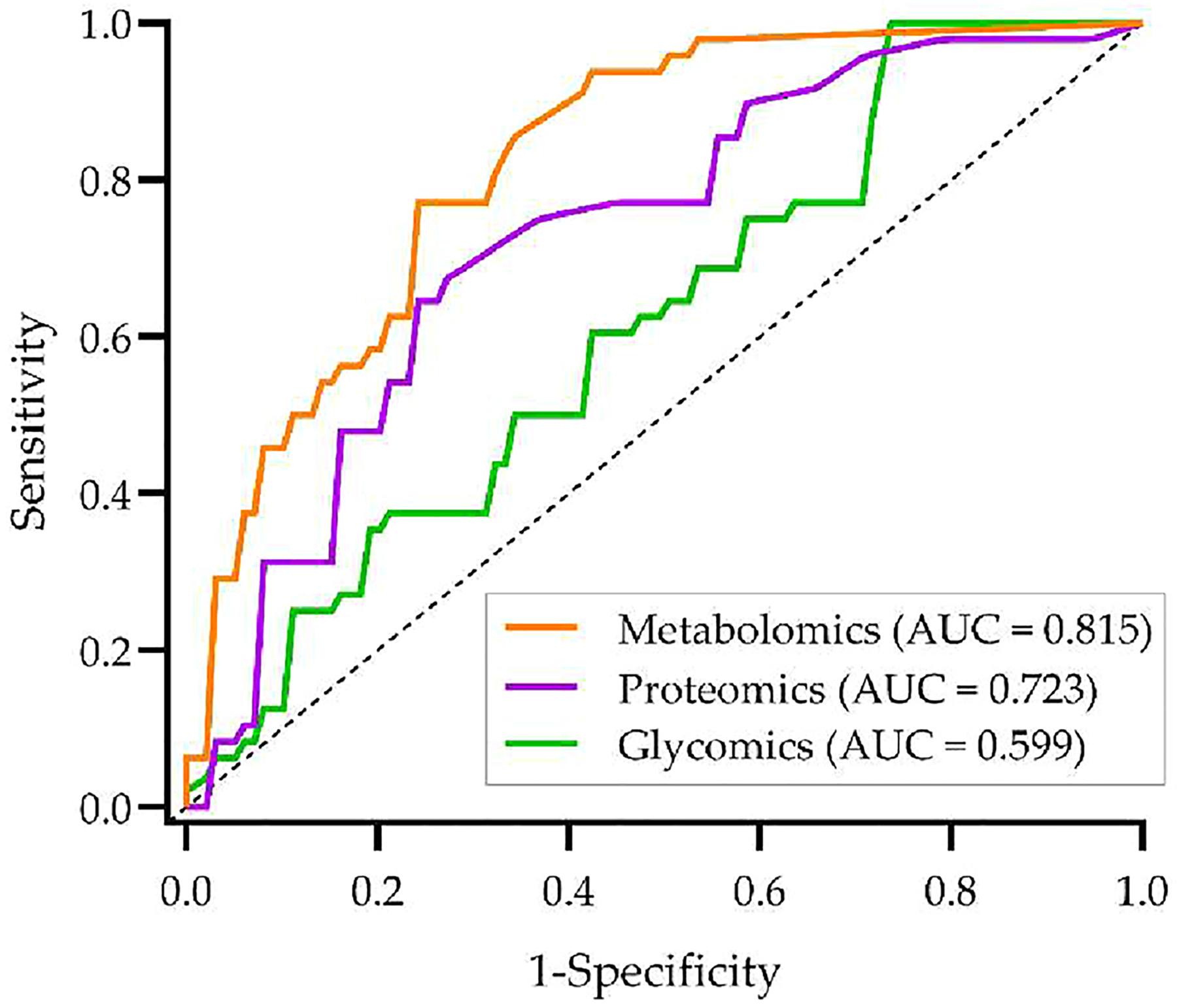
Receiver operating characteristic (ROC) curves for the top five features selected by Student’s *t* test from each omics data set separately.

Table 2 summarizes the number of molecular features included in the targeted multiomics studies and those whose levels changed statistically significantly in HCC vs CIRR (*p* < 0.05) by Student’s *t* test. Table 3 presents the top five features from each targeted omics study analyzed separately using Student’s *t* test. Figure 2 presents the receiver operating characteristic (ROC) curves and AUCs based on a logistic regression model with 5-fold cross validation applied on the top five features selected within each omics study. The top five features selected from the metabolomics study achieved the highest discriminative performance, with an AUC of 0.815.

Table 4 presents the top five multiomics features selected by seven methods (SelectKBest, Elastic Net, SVM-RFE, Transformer-RFE, RF, MOINER, and MOGONET) along with their disease classification accuracy and AUC. The features for the latter three methods were ranked based on either VIP or SHAP values following disease classification. From the table, we see that glutamic acid is the most consistently identified feature, indicating its strong relevance for distinguishing HCC from cirrhosis. Glutamic acid is known to play a complex role in HCC development and progression. Elevated serum glutamate levels have been observed in chronic liver diseases (e.g., cirrhosis, hepatitis) and HCC. Components of glutamine metabolism, including glutamine synthetase, glutamate dehydrogenase, and metabolites, have been identified as potential biomarkers for HCC.^25^ Lactic acid, behenic acid, and three proteins (Serum amyloid P-component [P02743], Plasminogen [P00747], and Coagulation factor XIII B chain [P05160]) were ranked in the top five by more than one feature selection method. The three proteins that are produced in the liver and lactic acid have been associated with HCC or liver cirrhosis. However, no direct link between behenic acid and HCC has been reported.^26,27^

**Figure 3.**
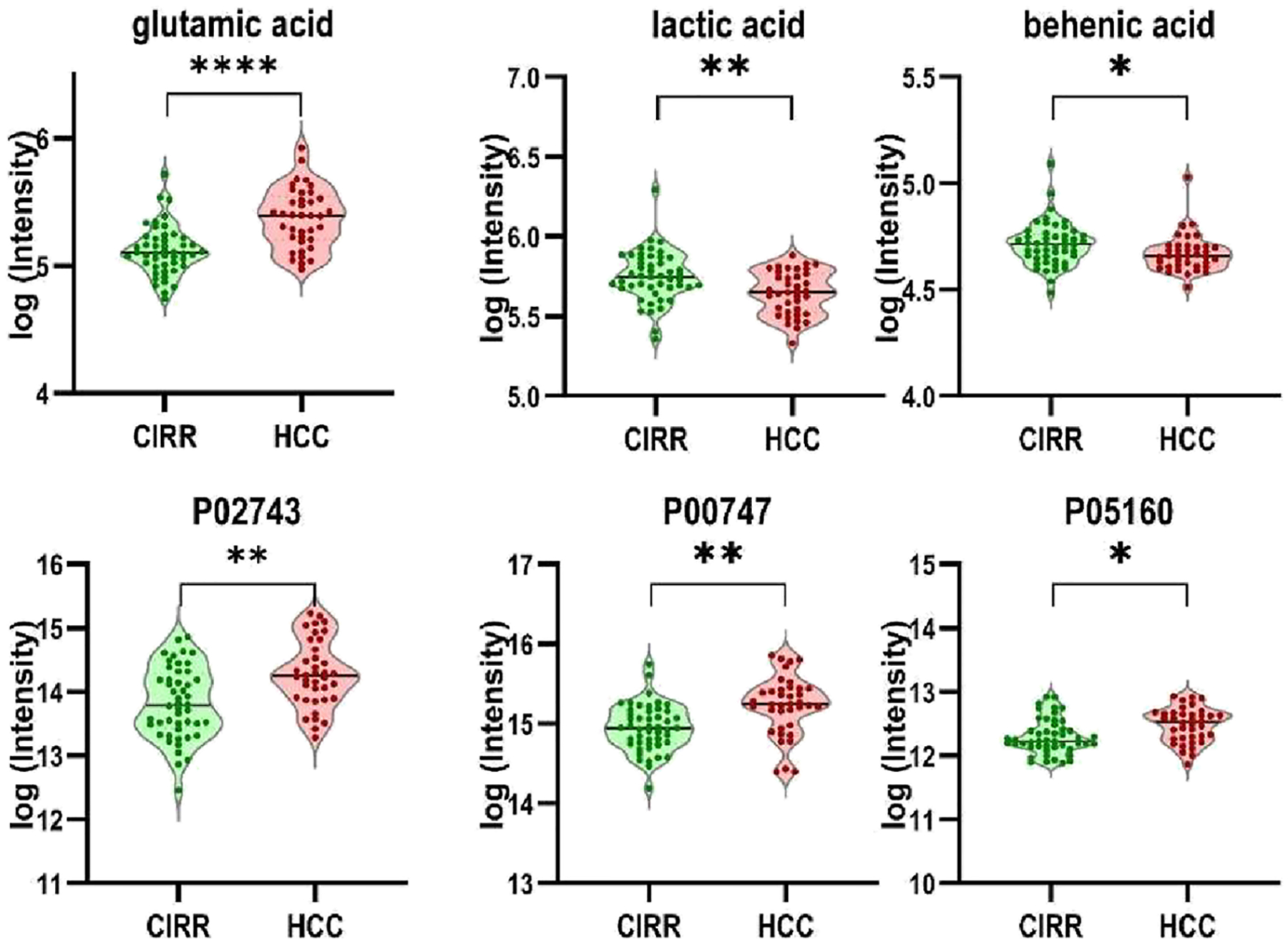
Multiomics features selected by more than one feature selection method.

Figure 3 depicts the log-transformed intensity values for the six most frequently selected multiomics features (glutamic acid, P02743, P00747, P05160, lactic acid, and behenic acid). All six features were statistically significantly altered in HCC vs CIRR (*p* < 0.05), with glutamic acid demonstrating the most pronounced separation. Combining all six features in a logistic regression model with 5-fold stratified cross-validation resulted in a classification accuracy of 0.744 ± 0.054 and an AUC of 0.852 ± 0.064.

**Figure 4.**
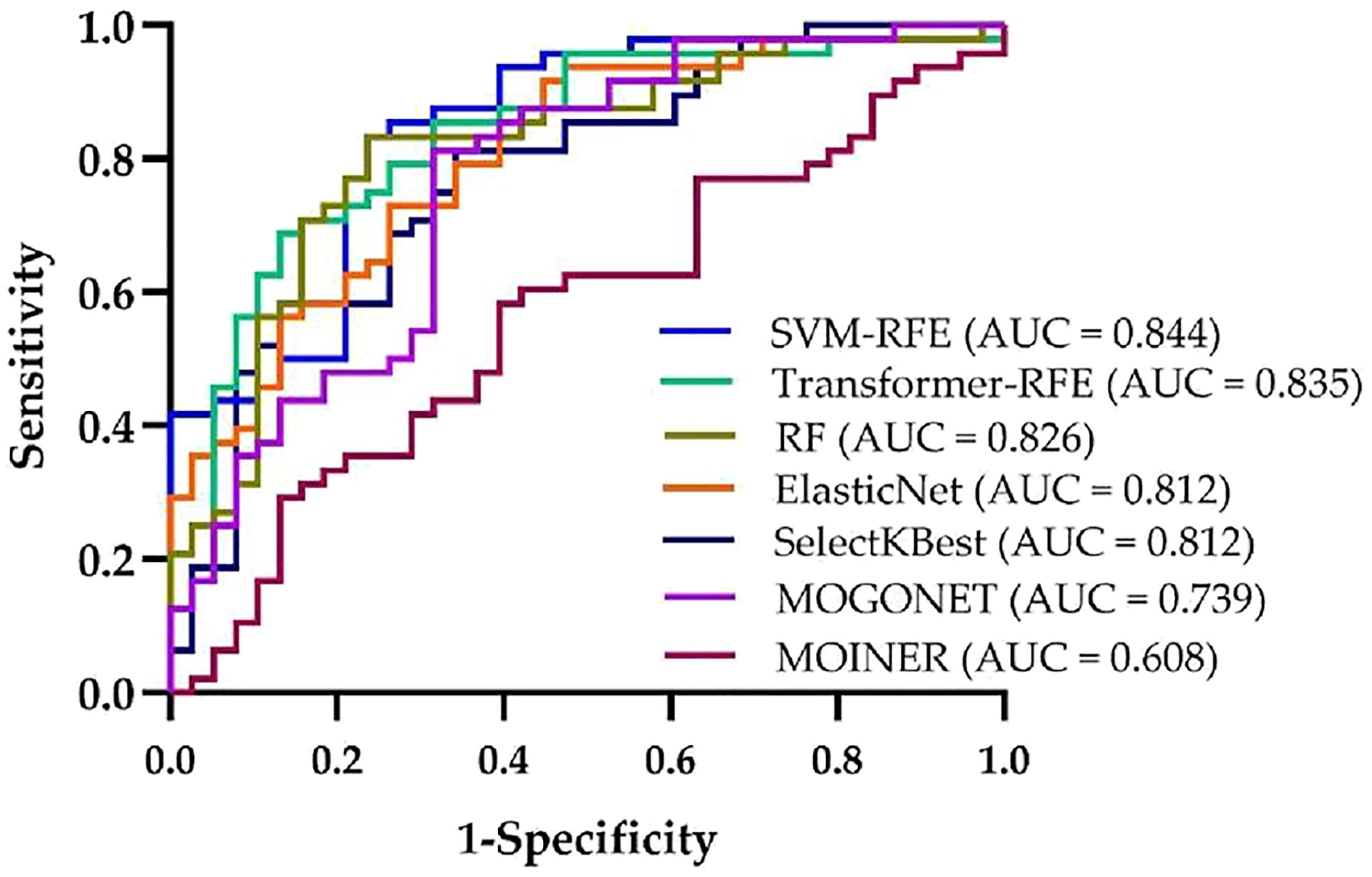
ROC curves of multiomics features selected by various methods and combined by logistic regression.

Figure 4 compares the classification performance of the top five multiomics features selected by each of the seven methods via ROC curves. The ROC curves are obtained by combining the top five features using logistic regression model and applying a 5-fold cross-validation. Among the methods, SVM-RFE and Transformer-RFE exhibited strong discriminative performance both in terms of classification accuracy and AUC values.

## CONCLUSIONS

Multiomics data acquired by analysis of serum or plasma samples from HCC cases and patients with liver cirrhosis in the Egyptian cohort identified key molecules that are associated with liver. The findings show that integrative analysis not only boosts predictive performance but also yields biologically meaningful multiomics signatures.

Deep learning frameworks such as MOINER and MOGONET generally achieve strong classification performance, but they are not inherently designed to perform feature selection during training. Instead, feature relevance is typically assessed post hoc using measures such as VIP or SHAP values, which quantify the contribution of each feature after the model has been fitted. This differs from approaches like recursive feature elimination (RFE), which iteratively remove or retain features by repeatedly retraining the classifier to assess the impact of different feature subsets. Extending such recursive retraining schemes to models like MOINER or MOGONET would be computationally prohibitive, given their architectural complexity and resource demands. This limitation underscores the need to adapt or augment deep learning models to enable principled feature selection, particularly in settings with limited multiomics sample sizes, as in this study. Furthermore, evaluation of the discovered multiomics features through independent cohorts is critical to identify robust biomarkers for HCC.

Our future work will focus on validating biomarker candidates chosen in this study via an independent cohort of larger sample size. Furthermore, we will continue to develop and optimize a transformer-based deep learning framework that possesses an inherent capability to fuse multiomics feature selection and disease classification into a single adaptive learning body. In this paper, we present a preliminary study on applying RFE with a transformer-based deep learning model as the base estimator. This integrated approach yields more promising results than other deep learning methods that perform disease classification and feature ranking as separate, sequential steps.

## Supplementary Material

Supplementary Material Table S1: metabolites, proteins, and N-linked glycans selected for targeted quantitation (XLSX)

The Supporting Information is available free of charge at https://pubs.acs.org/doi/10.1021/acs.jproteome.5c00741.

## Figures and Tables

**Table 1.** Characteristics of the Patients Whose Blood Samples Were Analyzed Using Multi-Omics Approaches

		HCC (*N* = 40)	CIRR (*N* = 49)	*p*-value
age	mean (SD)	53 (4)	53 (7)	0.3898
gender	male %	77.5	67.3	0.3474
	HCV Ab^+^	100%	100%	1
	HBs Ag^+^	0	6%	0.2492
etiology (%)	smoking	60%	53%	0.5165
	alcohol	0	0	1
MELD	mean (SD)	18.6 (7.7)	18.9 (7.1)	0.1328
	MELD < 10	20%	12%	0.3863
AFP	mean (SD)	932.9 (1318)		
HCC stage	stage 1	72.5%		1
	stage 2	15%		0.3101
	stage 3	5%		

**Table 2.** Number of Significant Features in Each Targeted Omics Dataset Based on Student’s *t*-Test

omics data set	# features	# features (*p* ≤ 0.05)
proteomics	100	39
glycomics	82	21
metabolomics	60	12

**Table 3.** Top Five Features Selected from Each Targeted Omics Dataset by Using Student’s *t*-Test

proteomics		glycomics		metabolomics	
protein	AUC	glycan	AUC	metabolite name	AUC
P00747	0.759	53100	0.62	glutamic acid	0.797
P02743	0.715	53111	0.608	lactic acid	0.695
P13598	0.737	43000	0.646	norvaline	0.671
P00751	0.721	53000	0.632	alpha-d-glucosamine 1-phosphate	0.672
P80108	0.724	43200	0.523	behenic acid	0.663
combined	0.723	combined	0.599	combined	0.815

**Table 4.** Top Five Features Were Selected from the Targeted Multi-Omics Data Using SelectKBest, Elastic Net, SVM-RFE, Transformer-RFE, RF, MOINER, and MOGONET^a^

	SelectKBest	Elastic Net	SVM-RFE	Transformer-RFE	RF	MOINER	MOGONET
multiomics features	glutamic acid	P05160	glutamic acid	glutamic acid	behenic acid	P27169	P0C0L4
	P02743	P13591	P02763	lactic acid	glutamic acid	73514	P22891
	P00747	glutamic acid	lactic acid	P10909	alpha-d-glucosamine 1-phosphate	64403	P01877
	P13598	P35858	P02743	33101	P05160	P02771	P02655
	P19320	lactic acid	behenic acid	P13796	P00747	P08519	glutamic acid
Accuracy	0.686	0.722	0.756	0.734	0.733	0.558	0.686
AUC	0.812	0.812	0.844	0.835	0.826	0.608	0.739

a Features identified by more than one method are shown in bold. Disease classification performance (accuracy and area under the ROC curve, AUC) was evaluated using a logistic regression model with five-fold cross-validation.

## Data Availability

Data generated in this work are available in the ProtemeXchange Consortium via the PRIDE partner repository (PXD001171), PeptideAtlas (PASS00542), and MetaboLights (MTBLS19 and MTBLS105).
